# Low skeletal muscle radiodensity and neutrophil-to-lymphocyte ratio as predictors of poor outcome in patients with COVID-19

**DOI:** 10.1038/s41598-022-20126-6

**Published:** 2022-09-20

**Authors:** Daniela M. H. Padilha, Maria C. S. Mendes, Fabiana Lascala, Marina N. Silveira, Lara Pozzuto, Larissa A. O. Santos, Lívia D. Guerra, Rafaella C. L. Moreira, Sandra R. Branbilla, Ademar D. C. Junior, Mateus B. O. Duarte, Maria L. Moretti, José B. C. Carvalheira

**Affiliations:** 1 Division of Oncology, Department of Anesthesiology, Oncology and Radiology, School of Medical Sciences, University of Campinas, Rua Vital Brasil, 80, Cidade Universitária, Campinas, SP Zip Code: 13.083-888 Brazil; 2 Department of Internal Medicine, School of Medical Sciences, University of Campinas, Campinas, SP Brazil; 3 Hematology and Oncology Clinics, Cancer Hospital of Cascavel, União Oeste de Estudos E Combate Ao Câncer (UOPECCAN), Cascavel, PR Brazil

**Keywords:** Infectious diseases, Respiratory tract diseases, Prognostic markers

## Abstract

Inflammatory states and body composition changes are associated with a poor prognosis in many diseases, but their role in coronavirus disease 2019 (COVID-19) is not fully understood. To assess the impact of low skeletal muscle radiodensity (SMD), high neutrophil-to-lymphocyte ratio (NLR) and a composite score based on both variables, on complications, use of ventilatory support, and survival in patients with COVID-19. Medical records of patients hospitalized between May 1, 2020, and July 31, 2020, with a laboratory diagnosis of COVID-19 who underwent computed tomography (CT) were retrospectively reviewed. CT-derived body composition measurements assessed at the first lumbar vertebra level, and laboratory tests performed at diagnosis, were used to calculate SMD and NLR. Prognostic values were estimated via univariate and multivariate logistic regression analyses and the Kaplan–Meier curve. The study was approved by the local Institutional Review Board (CAAE 36276620.2.0000.5404). A total of 200 patients were included. Among the patients assessed, median age was 59 years, 58% were men and 45% required ICU care. A total of 45 (22.5%) patients died. Multivariate logistic analysis demonstrated that a low SMD (OR 2.94; 95% CI 1.13–7.66, *P* = 0.027), high NLR (OR 3.96; 95% CI 1.24–12.69, *P* = 0.021) and both low SMD and high NLR (OR 25.58; 95% CI 2.37–276.71, *P* = 0.008) combined, were associated with an increased risk of death. Patients who had both low SMD and high NLR required more mechanical ventilation (*P* < 0.001) and were hospitalized for a longer period (*P* < 0.001). Low SMD, high NLR and the composite score can predict poor prognosis in patients with COVID-19, and can be used as a tool for early identification of patients at risk. Systemic inflammation and low muscle radiodensity are useful predictors of poor prognosis, and the assessment of these factors in clinical practice should be considered.

## Introduction

The coronavirus disease 2019 (COVID-19) pandemic has imposed significant stress on health systems worldwide. Just over 1 year after the outbreak in March 11, 2020, when the WHO declared the global pandemic, more than 4 million people had died due to SARS-CoV-2 infection^1^. Recent advances in the development of vaccines and anti-inflammatory treatments^2,3^ have dramatically changed the scenario of COVID-19 and offer hope for the future. Vaccines alone, however, are not enough to control the COVID-19 pandemic^4^. Therefore, multiple strategies are crucial to overcoming the pandemic. Since the earliest reports, there has been clear evidence of the association of comorbidities and inflammatory states with severity of COVID-19^5^. The most notable of these comorbidities include aging, diabetes, obesity, hypertension, and cancer, because they have inflammation and changes in body composition as common features.

Inflammation is the core of the pathophysiology of COVID-19^5,6^, while serum and hematologic biomarkers provide most of the evidence confirming the disease. White blood cell count, C-reactive protein, procalcitonin, erythrocyte sedimentation rate, interleukin-6, and interleukin-10^7^ are the more prominent biomarkers. The neutrophil-to-lymphocyte ratio (NLR) is an established biomarker of systemic inflammation in the literature. This biomarker has previously been associated with outcomes of cardiovascular conditions^8^, cancer^9^, and sepsis^10^. A meta-analysis has shown that this biomarker is also positively associated with COVID-19 outcomes^11^.

Myosteatosis, assessed by skeletal muscle radiodensity (SMD) on computed tomography (CT), is defined as an abnormal distribution of inter- and intramyocellular adipose tissue associated with reduced muscle quality, physical fitness, and muscle function^12^. Low SMD is correlated with higher rates of weight loss, increased systemic inflammatory response, and insulin resistance^13^. Myosteatosis is now considered a different entity from sarcopenia and is used as a prognostic marker in several diseases such as cancer, hepatopathy and, more recently, COVID-19^14–16^. Although sarcopenia and myosteatosis often co-occur, they are not mutually inclusive, where the additive effects of both parameters on muscle can be predictive of significantly poorer outcomes^17^.

Interestingly, in an assessment of hospitalized patients with COVID-19, Yang^18^ found an association between SMD and severity of COVID-19, while McGovern^19^ identified an association of mortality with visceral adipose tissue and skeletal muscle index but not with SMD. Identifying factors that impact the severity of COVID-19 is imperative, and risk scores are being developed to predict an inpatient’s chance of developing a critical illness^20,21^. This may help to develop new clinical tools for optimizing COVID-19 treatment, reducing the negative impact of the disease on health systems. Therefore, SMD, NLR, and a composite score based on both these variables, were evaluated as prognostic risk stratification factors for patients with COVID-19 at hospital admission.

## Methods

### Study population

Patients hospitalized at the Clinicas Hospital of the University of Campinas (UNICAMP) between May 1, 2020, and July 31, 2020, were retrospectively enrolled in the present analytical observational cohort. The inclusion criteria were as follows: (a) testing positive on the SARS-CoV-2 reverse transcriptase-polymerase chain reaction (RT-PCR) assay; (b) having a CT scan at the first lumbar vertebra (L1) level available on the hospital´s electronic system at the time of COVID-19 diagnosis; and (c) having the date of hospital discharge or death available in the medical record. Patients who refused to participate in the study, had contrast-enhanced or suboptimal CT image quality, or lacked data on clinical variables of interest were excluded.

### Data collection

Demographic, clinical, and biochemical data were collected from medical records. Information were recorded on hospitalization, date of first symptoms, and time from first symptoms to admission (fever, cough, myalgia, fatigue, diarrhea, nausea or vomiting, dyspnea, anosmia, dysgeusia, and headache), whether or not ICU care was required, ventilatory support (nasal catheter, non-rebreather mask, and mechanical ventilation), clinical complications (acute distress syndrome, acute cardiac injury, acute kidney injury, secondary infection, shock and pulmonary embolism), length of stay (LOS)—defined as length of time between date of hospitalization, either in ICU or general ward, and discharge or death, and outcome (death or discharge). Laboratory data for the period closest to the date of diagnosis were also collected. At the center in question, mechanical ventilation and endotracheal intubation is considered in the event of arterial oxygen saturation < 90% with fractional inspired oxygen (FiO_2_) > 0.60 and/or increased respiratory distress and/or reduced level of consciousness and/or shock (systolic blood pressure < 90 mmHg or mean arterial pressure < 65 mmHg or decrease of blood pressure > 40 mmHg and lactate > 1.6 mmol/L).

### Body composition assessment

Patients’ CT scan images performed at the time of COVID-19 diagnosis were obtained from an electronic medical image viewer. A single cross-sectional image nearest the inferior border of L1, using SliceOMatic V.5.0 software (Tomovision, Canada) was analyzed by a trained evaluator (M.C.S.M.) blinded to the patients´ clinical information^22^. The tissue Hounsfield Unit (HU) thresholds for skeletal muscle were − 29 to 150 HU. Skeletal muscle analysis included the psoas, latissimus dorsi, paravertebral muscles, quadratus lumborum, intercostal muscles, external oblique, internal oblique, rectus abdominis, and transversus abdominis^23^. Skeletal muscle area (SMA), skeletal muscle index (SMI) and skeletal muscle radiodensity (SMD) were investigated^24,25^. The cross-sectional area of SMs was measured in squared centimeters (cm^2^) and the skeletal muscle index (cm^2^/m^2^) was calculated as the total muscle area adjusted for height.

### Systemic inflammatory indexes

A complete blood count collected near the time of diagnosis was used to calculate NLR. The NLR was estimated by dividing neutrophils by absolute lymphocyte counts^26^. A high NLR indicates severe inflammation. A composite score was calculated using both SMD and NLR. Patients were allocated into the “Neither” group when they had a high SMD and low NLR, into the “Either” group when they had a low SMD or high NLR, and into the “Both” group when they had a low SMD and high NLR.

### Endpoints

The primary endpoint was 90-day mortality (calculated as the time interval between diagnosis and death from any cause). The secondary endpoints were LOS (time interval from admission to death or discharge) and need for ventilatory support.

### Statistical analysis

The Shapiro–Wilk test was employed to determine the normality of the distributions of variables. Categorical variables were expressed numerically (percentages), whereas continuous variables were expressed as means and standard deviations (SDs) for parametric distribution, or as medians and confidence intervals (95% CIs) for nonparametric distributions. Group comparisons were conducted using Student’s *t*-test, the Mann–Whitney U test, or Kruskal–Wallis test for continuous variables. Categorical variables were compared by applying the chi-squared test or Fisher’s exact test when appropriate.

SMD, SMI, SMA and NLR were categorized into low or high groups. The Youden index was used to determine the optimal cutoff values^27^. Cutoff points for SMD, SMI and SMA were sex specific. The impacts of low SMA, SMD, SMI and high NLR on the survival outcome were assessed alone or combined in a score evaluating neither, either or both risk factors (low SMD and high NLR) using univariate and multivariate Cox proportional-hazard regression analyses. Variables with *P* < 0.1 on the univariate analysis were included in the multivariate model. The Cox model was adjusted for age (continuous), BMI (continuous), one or more comorbidities (categorical), two or more comorbidities (categorical), creatinine (categorical), hemoglobin (categorical), alanine aminotransferase (categorical), sodium (categorical), and prothrombin time (categorical). Kaplan–Meier curves were plotted for graphical visualization. All data collected were stored using the electronic data collection tool Research Electronic Data Capture (REDCap)^28^. Statistical analyses were executed using Stata, version 12.0 (StataCorp LP, College Station, United States of America), and statistical significance was considered with a two-sided *P* < 0.05.

### Ethical approval

This study was approved by the local Institutional Review Board (Comitê de Ética em Pesquisa [CEP]) (CAAE: 36276620.2.0000.5404). All procedures observed the Declaration of Helsinki, and informed consent was obtained from all subjects.

## Results

### Patient characteristics

**Figure 1:**
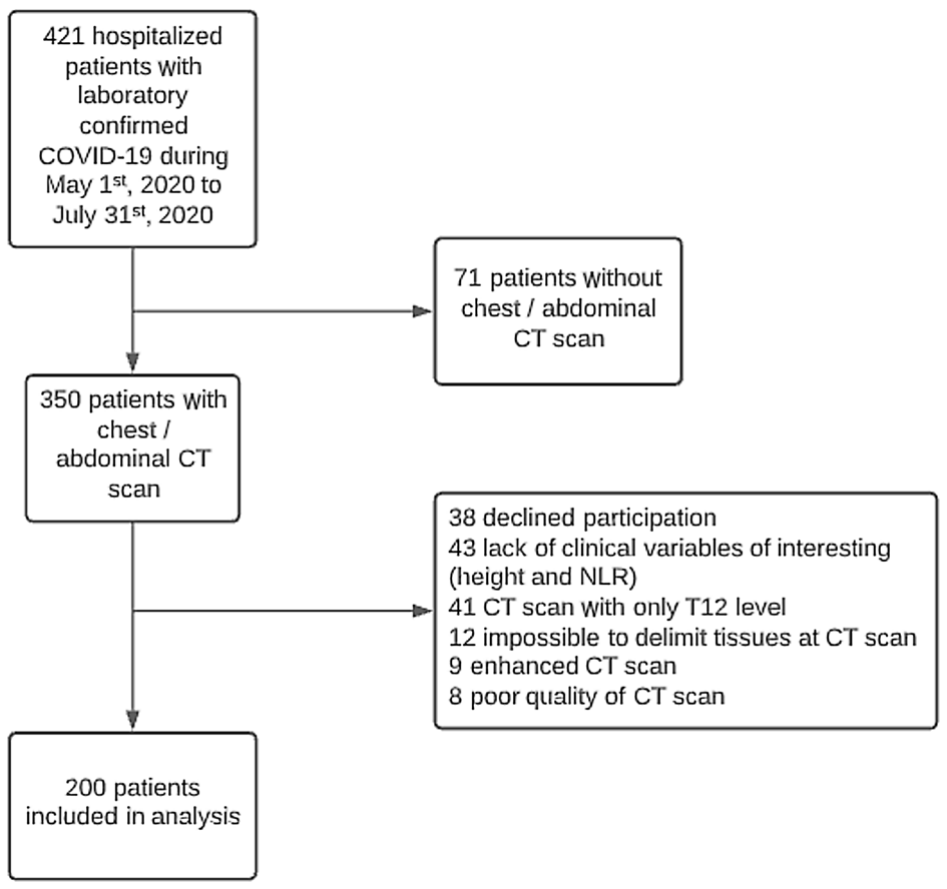
Flow-chart showing the patient recruitment process. Abbreviations: NLR: Neutrophil-to-lymphocyte ratio; CT: Computed tomography.

A total of 421 patients were hospitalized at the UNICAMP between May 1, 2020, and July 31, 2020, with a laboratory-confirmed SARS-CoV-2 infection. After the exclusion of 221 patients due to the absence of a CT scan at the L1 level, participation refusal, or the absence of clinical variables of interest, 200 patients were included in the analysis (Fig. 1). In total, 45 patients (22.5%) died during this period. The median time between CT scan and onset of symptoms was 7 days (IQR 4.5–10.0), and the median time between CT scan and hospitalization was 0 days (IQR 0.0–1.0).

The clinical and physical characteristics of patients are summarized in Table 1. Among the study population, median age was 59.1 years, 58.0% were male, 69.5% White/Caucasian and 59.5% had BMI < 30 kg/m^2^. Regarding comorbidities, the most frequent were hypertension (57.5%), diabetes (33.0%) and dyslipidemia (16.5%). Approximately 10% of patients had cancer, of which 17 (80.95%) were under treatment. Most of the hospitalized patients had ≥ 1 comorbidity (89%). The most frequent symptoms on admission were fever (66.5%), dyspnea (61.0%) and cough (59.0%), while 9.5% of patients were asymptomatic.

**Table 1:** Selected demographic and clinical characteristics, as well as laboratory findings according to skeletal muscle radiodensity (SMD) and neutrophil-to-lymphocyte ratio (NLR) of patients with COVID-19.

Characteristic	All-patients, n = 200	Skeletal muscle radiodensity (SMD)			Neutrophil-to-lymphocyte index (NLR)		
		Low, n = 71 Female: < 27.7 Male: < 35.5	High, n = 129 Female: > 27.7 Male: > 35.5	*P* value	Low, n = 72 < 4.2	High, n = 128 > 4.2	*P* value
Age, mean (SD)	59.1 (14.3)	66.0 (13.5)	55.6 (13.3)	** < 0.001**^**a**^	58.2 (12.1)	59.7 (15.4)	0.471^a^
**Sex, No (%)**							
Female	84 (42.0)	28 (39.4)	56 (43.4)	0.586^b^	26 (36.1)	58 (45.3)	0.206^b^
Male	116 (58.0)	43 (60.6)	73 (56.9)		46 (63.9)	70 (54.7)	
**BMI, No (%)**							
< 30	119 (59.5)	49 (69.0)	70 (54.3)	**0.042**^**b**^	33 (45.8)	86 (67.2)	**0.003**^**b**^
$\ge$ 30	81 (40.5)	22 (31.0)	59 (45.7)		39 (54.2)	42 (32.8)	
**Race, No (%)**							
African American	15 (7.5)	3 (4.2)	12 (9.3)	0.250^c^	10 (13.9)	5 (3.9)	**0.018**^**b**^
White/Caucasian	139 (69.5)	48 (67.6)	91 (70.5)		43 (59.7)	96 (75.0)	
African + Caucasian	46 (23.0)	20 (28.2)	26 (20.2)		19 (26.4)	27 (21.1)	
**Smoking, No (%)**							
Never	109 (64.1)	33 (54.1)	76 (69.7)	0.108^c^	40 (64.5)	69 (63.9)	0.609^c^
Former smoker (more than 5 years)	40 (23.5)	19 (31.1)	21 (19.3)		17 (27.4)	23 (21.3)	
Former smoker (less than 5 years)	13 (7.7)	7 (11.5)	6 (5.5)		3 (4.9)	10 (9.3)	
Active smoker	8 (4.7)	2 (3.3)	6 (5.5)		2 (3.2)	6 (5.5)	
**Comorbidities, No (%)**							
Hypertension	115 (57.5)	‘51 (71.8)	64 (49.6)	**0.002**^**b**^	38 (52.8)	77 (60.2)	0.311^b^
Dyslipidemia	33 (16.5)	11 (15.5)	22 (17.1)	0.776^b^	13 (18.1)	20 (15.6)	0.657^b^
Emphysema	10 (5.0)	3 (4.2)	7 (5.4)	1.000^c^	5 (6.9)	5 (3.9)	0.500^c^
Chronic Kidney Disease	28 (14.0)	11 (15.5)	17 (13.2)	0.652^b^	5 (6.9)	23 (18.0)	**0.034**^**c**^
Congestive heart failure	14 (7.0)	5 (7.0)	9 (7.0)	1.000^c^	3 (4.2)	11 (8.6)	0.387 ^c^
Coronaropathy	21 (10.5)	9 (12.7)	12 (9.3)	0.456^b^	9 (12.5)	12 (9.4)	0.489^b^
Stroke	7 (3.5)	4 (5.6)	3 (2.3)	0.248^c^	1 (1.4)	6 (4.7)	0.425^c^
Chronic liver Disease	7 (3.5)	2 (2.8)	5 (3.9)	1.00^c^	5 (6.9)	2 (1.6)	0.101^c^
Autoimmune Rheumatic Diseases	5 (2.5)	1 (1.4)	4 (3.1)	0.657^c^	2 (2.8)	3 (2.3)	1.000^c^
Cancer	21 (10.5)	9 (12.7)	12 (9.3)	0.456^b^	7 (9.7)	14 (11.0)	1.000^c^
Diabetes	66 (33.0)	30 (42.3)	36 (27.9)	**0.039**^**b**^	23 (31.9)	43 (33.6)	0.812^b^
1 or more comorbidity	178 (89.0)	66 (93.0)	112 (86.8)	0.184^b^	66 (91.7)	112 (87.5)	0.366^b^
2 or more comorbidities	129 (64.5)	53 (74.7)	76 (58.9)	**0.026**^**b**^	39 (54.2)	90 (70.3)	**0.022**^**b**^
**Symptoms on admission, No (%)**							
Fever	133 (66.5)	42 (59.2)	91 (70.5)	0.103^b^	47 (65.3)	86 (67.19)	0.366
Cough	118 (59.0)	40 (56.3)	78 (60.5)	0.570^b^	42 (58.3)	76 (59.38)	0.886
Myalgia	68 (34.0)	20 (28.2)	48 (37.2)	0.197^b^	28 (38.9)	40 (31.25)	0.274
Fatigue	24 (12.0)	5 (7.0)	19 (14.7)	0.171^c^	11 (15.3)	13 (10.16)	0.285
Diarrhea	39 (19.5)	12 (16.9)	27 (20.9)	0.491^b^	15 (20.8)	24 (18.75)	0.721
Nausea or vomiting	38 (19.0)	9 (12.7)	29 (22.5)	0.091^b^	13 (18.1)	25 (19.53)	0.798
Dyspnea	122 (61.0)	42 (59.2)	80 (62.0)	0.691^b^	44 (61.1)	78 (60.94)	0.981
Anosmia	28 (14.0)	3 (4.2)	25 (19.4)	**0.003**^**c**^	13 (18.1)	15 (11.72)	0.215
Dysgeusia	22 (11.0)	4 (5.6)	18 (14.0)	0.098^c^	12 (16.7)	10 (7.81)	0.055
Headache	39 (19.5)	10 (14.1)	29 (22.5)	0.152^b^	14 (19.4)	25 (19.53)	0.988
Asymptomatic	19 (9.5)	8 (11.3)	11 (8.5)	0.616^c^	7 (9.7)	12 (9.38)	1.00
**Laboratory findings, No (%)**							
Hemoglobin (M: < 14 g/dL; F: < 12 g/dL)	90 (45.0)	46 (64.8)	44 (34.1)	** < 0.001**^**b**^	20 (27.8)	70 (54.7)	** < 0.001**^**b**^
ALT (> 35 U/L)	66 (34.0)	14 (20.6)	52 (41.3)	**0.004**^**b**^	25 (36.8)	41 (32.5)	0.553^b^
Sodium (< 135 mEq/L)	58 (29.6)	26 (36.6)	32 (25.6)	0.104^b^	18 (25.4)	40 (32.0)	0.327^b^
Creatinine (> 1.2 mg/dL)	64 (32.2)	33 (47.1)	31 (24.0)	**0.001**^**b**^	13 (18.1)	51 (40.2)	**0.001**^**b**^
Prothrombin time (> 14 s)	26 (13.5)	13 (19.1)	13 (10.4)	0.090^b^	5 (7.4)	21 (16.8)	0.079^c^
D-dimer (> 500 mg/mL)	146 (86.4)	56 (91.8)	90 (83.3)	0.123^b^	45 (79.0)	101 (90.2)	**0.044**^**b**^
CRP (> 3 mg/L)	179 (96.2)	64 (98.5)	115 (95.0)	0.243^b^	62 (95.4)	117 (96.7)	0.655^b^
Glycemia (> 100 mg/dL)	151 (76.7)	55 (78.6)	96 (75.6)	0.636^b^	49 (70.0)	102 (80.3)	0.101^b^

Missing data: ALT: 7; sodium: 4; creatinine: 1; prothrombin time: 7; D-dimer: 31; CRP: 14; and glycemia: 3

*ALT* alanine aminotransferase, *BMI* Body mass index, *CRP* C-reactive protein, *SD* standard deviation.

^a^Student’s *t*-test.

^b^chi-squared test.

^c^Fisher’s exact test.

Bold indicates *P* value is statistcally significant.

### Demographics, clinical characteristics, laboratory findings, and complications according to SMD and NLR of COVID-19 patients

**Figure 2:**
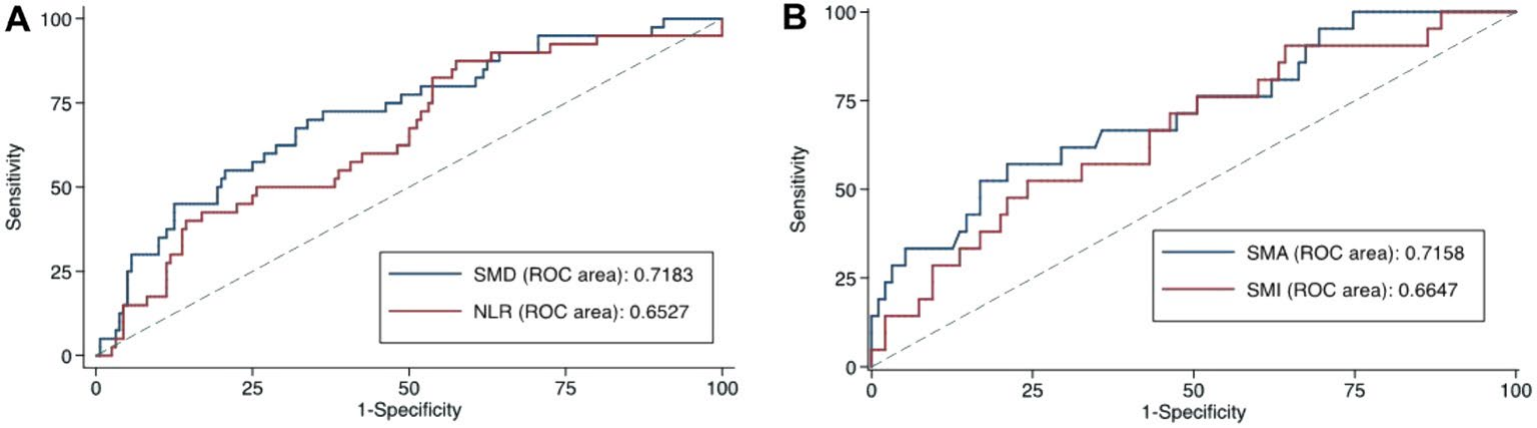
Youden index for (**A**) skeletal muscle radiodensity and neutrophil‐to‐lymphocyte ratio; and (**B**) skeletal muscle area and skeletal muscle index for patients with COVID-19. Abbreviations: SMA: Skeletal muscle area; SMD: Skeletal muscle radiodensity; SMI: Skeletal muscle index; NLR: Neutrophil-to-lymphocyte ratio; ROC: Receiver operating characteristic curve.

The cutoff values established using the Youden index were: (a) low SMD < 27.7 HU for females and < 35.5 HU for males; (b) low SMA < 118.9 m^2^ for females and < 119.9 m^2^ for males; (c) low SMI < 57.9 cm^2^/m^2^ for females and < 42.0 cm^2^/m^2^ for males; and (d) high NLR > 4.2. (Fig. 2). The group with low SMD was older (*P* < 0.001), had significantly lower BMI (*P* = 0.042), more hypertension (*P* = 0.002), more diabetes (*P* = 0.039) and ≥ 2 comorbidities (*P* = 0.026). This group also had significantly lower hemoglobin (*P* < 0.001), higher alanine aminotransferase (*P* = 0.004), and higher creatinine (*P* = 0.001) (Table 1). Patients with low SMD also had a higher prevalence of acute kidney injury (*P* = 0.005), shock (*P* = 0.001), and ≥ 2 complications (*P* = 0.026) during hospitalization (Table 2).

**Table 2:** Selected complications according to skeletal muscle radiodensity (SMD) and neutrophil-to-lymphocyte ratio (NLR) of patients with COVID-19.

Characteristic	All-patients, n = 200	Skeletal Muscle Radiodensity (SMD)			Neutrophil-to-lymphocyte index (NLR)		
		Low, n = 71 Female: < 27.7 Male: < 35.5	High, n = 129 Female: > 27.7 Male: > 35.5	*P* value	Low, n = 72 < 4.2	High, n = 128 > 4.2	*P* value
Acute distress syndrome	164 (82.0)	62 (87.3)	102 (79.1)	0.146^a^	53 (73.6)	111 (86.7)	**0.021**^a^
Acute cardiac injury	7 (3.5)	4 (5.6)	3 (2.3)	0.248^b^	1 (1.4)	6 (4.7)	0.425^b^
Acute kidney injury	43 (21.5)	23 (32.4)	20 (15.5)	**0.005**^a^	7 (9.7)	36 (28.1)	**0.002**^a^
Secondary infection	64 (32.0)	28 (39.4)	36 (27.9)	0.094^a^	15 (20.8)	49 (38.3)	**0.011**^a^
Shock	45 (22.5)	25 (35.2)	20 (15.5)	**0.001**^a^	8 (11.1)	37 (28.9)	**0.004**^a^
Pulmonary embolism	12 (6.0)	6 (8.5)	6 (4.7)	0.353^a^	2 (2.8)	10 (7.8)	0.218^b^
1 or more complications	178 (89.0)	66 (93.0)	112 (86.8)	0.184^a^	66 (91.7)	112 (87.5)	0.366^a^
2 or more complications	129 (64.5)	53 (74.7)	76 (58.9)	**0.026**^a^	39 (54.2)	90 (70.3)	**0.022**^a^

^a^Chi-square test.

^b^Fisher’s exact test.

Bold indicates *P* value is statistcally significant.

Patients in the high NLR group had significantly lower BMI (*P* = 0.003), more chronic kidney disease (*P* = 0.034) and ≥ 2 comorbidities (*P* = 0.022). These patients also had lower hemoglobin (*P* < 0.001), higher creatinine (*P* = 0.001), and higher D-dimer (*P* = 0.044) (Table 1). Some complications were more frequent in the high NLR group, such as acute distress syndrome (*P* = 0.021), acute kidney injury (P = 0.002), secondary infection (*P* = 0.011), and shock (*P* = 0.004). More patients with high NLR had ≥ 2 complications (*P* = 0.022) (Table 2).

### Comparison of selected complications according to SMD and NLR composite score

**Figure 3:**
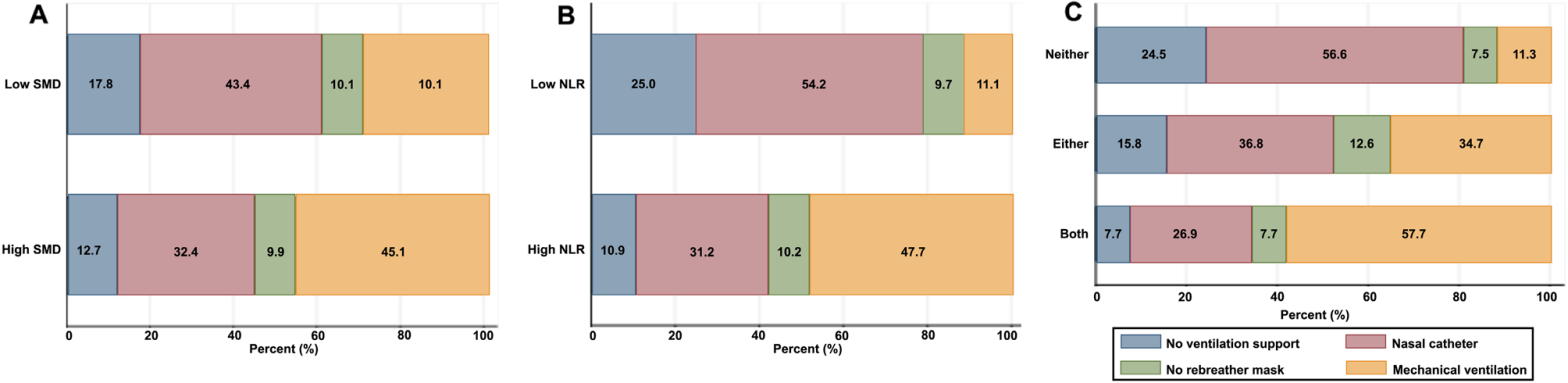
Supporting ventilatory use according to: (**A**) skeletal muscle radiodensity; (**B**) neutrophil-to-lymphocyte ratio; and (**C**) skeletal muscle radiodensity and neutrophil‐to‐lymphocyte ratio composite score of patients with COVID-19. Abbreviations: SMD: Skeletal muscle radiodensity; NLR: Neutrophil-to-lymphocyte ratio.

Median LOS was 12 days (IQR 6.0–25.0) for the overall population. Patients with low SMD had a longer LOS when compared to patients with high SMD (18.5 days, IQR 6.0–33.0 vs. 11.0 days, IQR 6–19.5; *P* < 0.001). The LOS was longer for patients with high NLR (15.0 days, IQR 9.0–29.0) compared to a low NLR (7 days, IQR 4.0–13.5), as well as for those in the Both group (19.0 days, IQR 8.0–33.0) compared to the Neither (7.0 days, IQR 4.0–11.0) and Either (14.0 days, IQR 7.0–29.0) (all *P* < 0.001). Regarding need for ventilatory support, the rates of mechanical ventilation were four times greater in patients with high NLR than in patients with low NLR (47.7% vs. 11.1%, *P* < 0.001). Patients in the Both group required more mechanical ventilation than patients in the Either or Neither groups (57.7% vs. 34.7% vs. 11.3%, *P* < 0.001) (Fig. 3).

Patients with both low SMD and high NLR had more complications, such as acute distress syndrome (*P* = 0.042), acute kidney injury (*P* < 0.001), secondary infection (*P* = 0.008), and shock (*P* < 0.001). Two or more complications was also more frequent among patients in the Both group (*P* = 0.010) (Table 3).

**Table 3:** Selected COVID-19 complications according to skeletal muscle radiodensity (SMD) and neutrophil-to-lymphocyte ratio (NLR) composite score of patients with COVID-19.

Characteristic	Neither SMD high and NLR low	Either SMD low or NLR high	Both SMD low and NLR high	*P* value
Acute distress syndrome	39 (73.6)	77 (81.1)	48 (92.3)	**0.042**^**a**^
Acute cardiac injury	0 (0.0)	4 (4.2)	3 (5.8)	0.255^b^
Acute kidney injury	5 (9.4)	17 (17.9)	21 (40.4)	** < 0.001**^b^
Secondary infection	8 (15.1)	35 (36.8)	21 (40.4)	**0.008**^**a**^
Shock	6 (11.3)	16 (16.8)	23 (44.2)	** < 0.001**^b^
Pulmonary embolism	2 (3.8)	4 (4.2)	6 (11.5)	0.180^b^
1 or more complications	49 (92.5)	80 (84.2)	49 (94.2)	0.115^a^
2 or more complications	26 (49.1)	63 (66.3)	40 (76.9)	**0.010**^**a**^

^a^chi-square test.

^b^Fisher’s exact test.

Bold indicates *P* value is statistcally significant.

### Association of body composition characteristics, NLR, and composite score with outcomes

**Figure 4:**
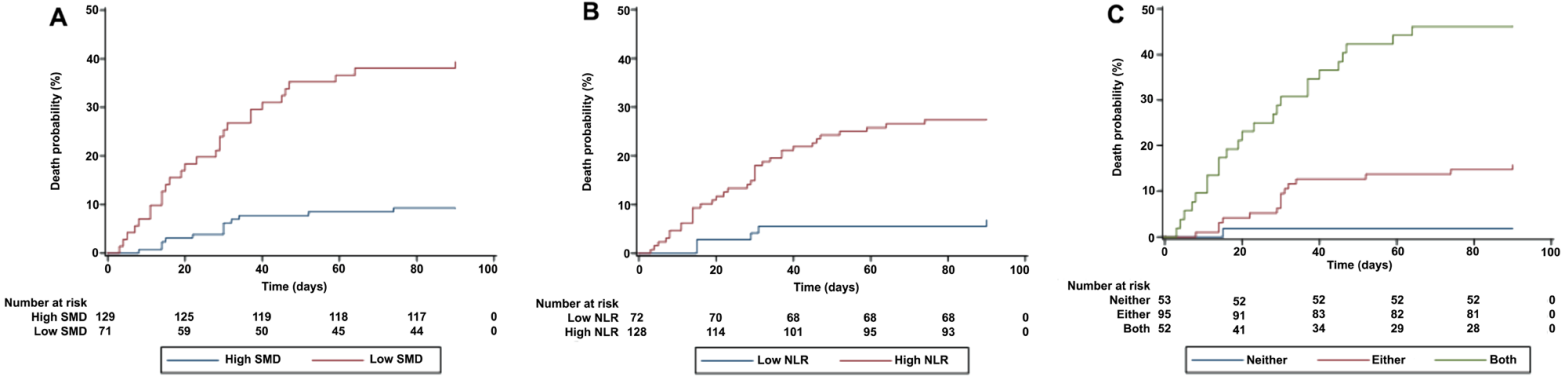
Overall survival according to: (**A**) skeletal muscle radiodensity; (**B**) neutrophil‐to‐lymphocyte ratio; and (**C**) skeletal muscle radiodensity and neutrophil‐to‐lymphocyte ratio composite score of patients with COVID-19. Abbreviations: SMD: Skeletal muscle radiodensity; NLR: Neutrophil-to-lymphocyte ratio.

According to the Kaplan–Meier curves, there was an increased probability of death in patients with a low SMD alone, high NLR alone or combination of the two (Fig. 4). Univariate logistic regression demonstrated that low SMA in males (OR 5.0; 95% CI 1.85–13.53, *P* = 0.002); low SMD in all patients, irrespective of sex (OR 6.35; 95% CI 2.97–13.59, *P* < 0.001); high NLR (OR 5.04; 95% CI 1.88–13.55, *P* = 0.001); and composite scores in the Either and Both groups (OR 9.75; 95% CI 1.25–76.05, *P* = 0.030 and OR 44.57; 95% CI 5.72–347.08, *P* < 0.001, respectively) were all associated with an increased risk of death. Multivariate logistic regression analysis revealed that males with low SMA had an increased risk of death (OR 8.33; 95% CI 2.21–31.32, *P* = 0.002). In all patients, low SMD (OR 3.33; 95% CI 1.28–8.65, *P* = 0.014) and high NLR (OR 4.39; 95% CI 1.40–13.77, *P* = 0.11) were also associated with an increased risk of death. Patients with either a low SMD or high NLR had a ten-fold greater risk of death (OR 10.42; 95% CI 1.03–105.21, *P* = 0.047), while patients with both a low SMD and high NLR had a higher risk of death (OR 28.88; 95% CI 2.77–300.77, *P* = 0.005) (Table 4).

**Table 4:** Univariate and multivariate Cox regression analyses of death probability according to low muscularity, low muscle radiodensity, high neutrophil-to-lymphocyte ratio and composite score in patients with COVID-19.

Characteristic	Univariate analysis			Multivariate analysis		
	OR	95% CI	*P* value	OR	95% CI	*P* value
**Low SMA**						
All Patients	2.04	0.999–4.15	0.050	2.02	0.80–5.12	0.136
Female	0.54	0.17–1.70	0.293	0.53	0.06–5.02	0.581
Male	5.00	1.85–13.53	**0.002**	8.33	2.21–31.32	**0.002**
**Low SMI**						
All Patients	0.80	0.24–2.59	0.704	1.00	0.22–4.48	0.998
Female	0.17	0.026–1.10	0.063	0.217	0.00–78.49	0.612
Male	2.62	0.32–21.48	0.370	2.68	0.23–31.91	0.435
**Low SMD**						
All Patients	6.35	2.97–13.59	** < 0.001**	3.33	1.28–8.65	**0.014**
Female	10.2	3.13–33.19	** < 0.001**	14.87	1.42–155.64	**0.024**
Male	4.55	1.66–12.46	**0.003**	2.84	0.83–9.74	0.096
High NLR	5.04	1.88–13.55	**0.001**	4.39	1.40–13.77	**0.011**
**Composite score**						
Either (low SMD or high NLR)	9.75	1.25–76.05	**0.030**	10.42	1.03–105.21	**0.047**
Both (low SMD and high NLR)	44.57	5.72–347.08	** < 0.001**	28.88	2.77–300.77	**0.005**

The Cox model was adjusted for age (continuous), BMI (continuous), ≥ 1 comorbidities (categorical), ≥ 2 comorbidities (categorical), creatinine (categorical), hemoglobin (categorical), alanine aminotransferase (categorical), sodium (categorical), and prothrombin time (categorical) and covariate missing data was completed with the variable median.

*CI* confidence interval, *OR* odds ratio, *NLR* neutrophil-to-lymphocyte ratio, *SMA* skeletal muscle area, *SMD* skeletal muscle radiodensity, *SMI* skeletal muscle index.

Bold indicates *P* value is statistcally significant.

## Discussion

The present study is one of the largest evaluating body composition with CT in a Latin American population hospitalized with COVID-19. A low SMD, high NLR or a composite score with both variables, proved independent predictors of the need for ventilatory support, LOS and death.

Approximately 60% of patients in the cohort had hypertension and 33% had diabetes, rates consistent with other studies involving hospitalized COVID-19 patients^29,30^. However, 60% had BMI < 30 kg/m^2^. Obesity was more prevalent in most previous studies than in the present population^29^.

The gold standard for assessing body composition is using a CT image at L3 level^22^. However, according to Derstine BA et al., L2, L4, L5, L1, T12, T11 and T10 levels (in this order of preference) may be used instead when L3 level is not available^31^. Therefore, skeletal muscle at the L1 level was evaluated, as abdominal CT scans are not performed in the routine assessment of patients with COVID-19.

Low SMD assessed by CT images is a prognostic factor of poor outcome in critically-ill and non-critically ill patients^32,33^, including in COVID-19 cases^16^. In ICU patients, low SMD at ICU admission was independently associated with higher 6-month mortality in non-COVID-19 mechanically-ventilated patients^34^, while preserved SMD at the commencement of venovenous extracorporeal membrane oxygenation was associated with improved ICU survival in non-COVID-19 patients^35^. More recently, low SMD has been associated with higher risk of ICU mortality in COVID-19 patients^36^. In the present cohort, by contrast, body composition was assessed at hospital admission to stratify risk factors of poor prognosis. Results revealed that low SMD was associated with more clinical complications (acute kidney injury, shock, and ≥ 2 complications), a greater need for mechanical ventilation, and death.

One of the factors that can lead to reduced SMD is the infiltration of fat into the muscle, referred to as myosteatosis. It has recently been reported that lipid accumulation, both intramyocellular and around the muscle (extramyocellular), results in the low muscle radiodensity recognized in patients with cancer^37^. Insulin resistance and inflammation are the leading causes of muscular lipid accumulation^38^. However, the pathophysiology of this process in patients with COVID-19 is not fully understood^39^. One hypothesis is that muscle injury in an inflammatory environment can trigger adipogenic and fibrogenic cells resident in muscle tissue, replacing muscle with fibrous and fatty tissues^40,41^.

**Figure 5:**
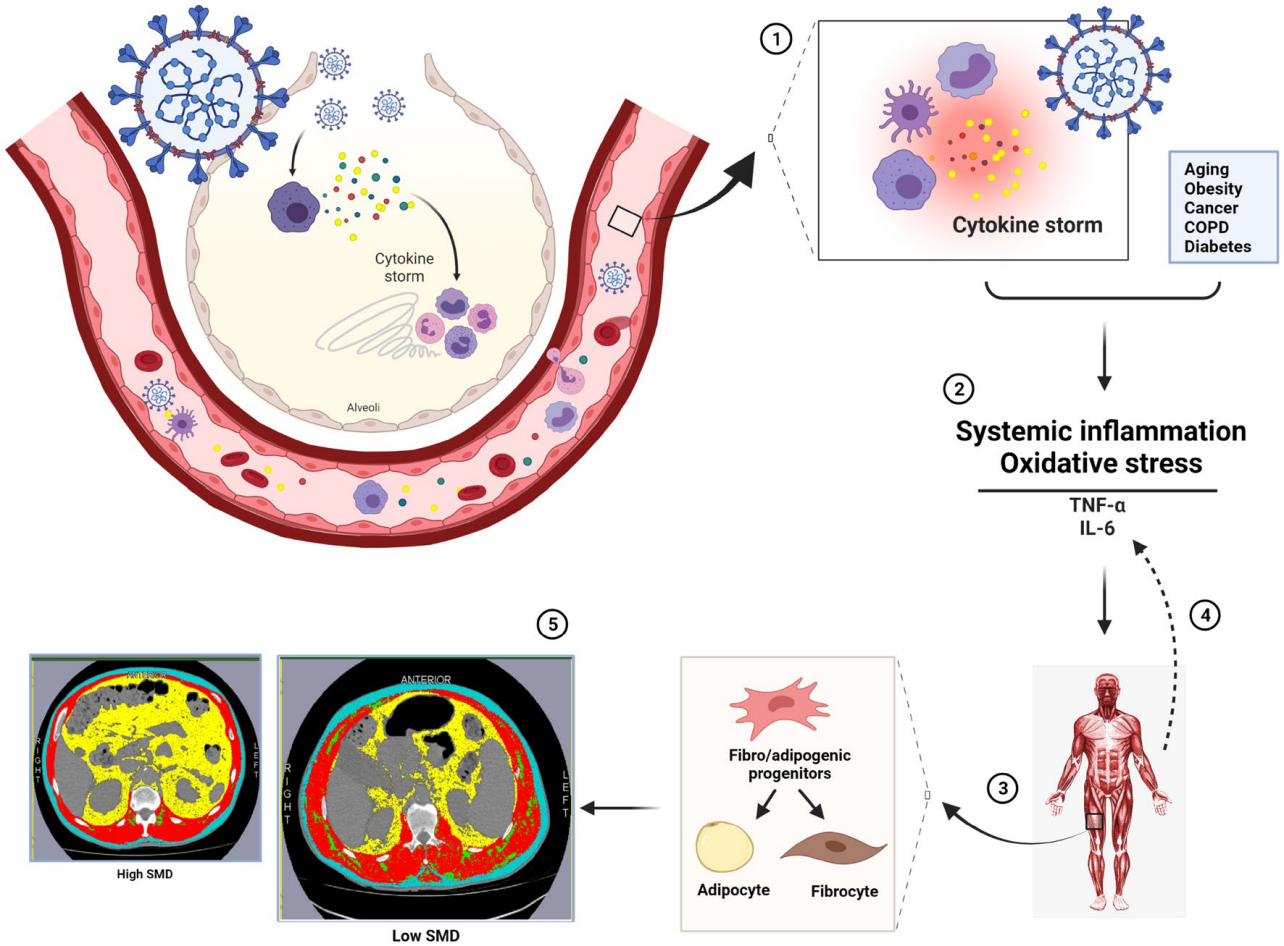
Proposed pathophysiology for low muscle radiodensity in COVID-19. 1. COVID-19 infection and increased inflammatory response (cytokine storm); 2. Systemic inflammation and oxidative stress due to COVID-19 and underlying comorbidities with high levels of circulating cytokines (e.g., IL-6 and TNF-α) and high NLR; 3. Fibro-/adipogenic progenitors in skeletal muscle differentiate into fibrocytes and adipocytes; 4. Myositis increases cytokine release; and 5. Increase in intramuscular adipose tissue leading to low SMD. Abbreviations: COPD: Chronic obstructive pulmonary disease; IL-6: Interleukin 6; NLR: Neutrophil‐to‐lymphocyte ratio; SMD: Skeletal muscle radiodensity; TNF-α: Tumor necrosis factor alpha.

Most patients with severe COVID-19 exhibit substantially elevated serum levels of pro-inflammatory cytokines, including IL-6^42^. IL-6 is an essential mediator of muscle wasting^43,44^. IL-6 induces the Janus kinase (JAK)/signal transducer and activator of transcription (STAT) pathways, which can trigger opposite effects. IL-6/JAK/STAT3 signaling can promote muscle hypertrophy, inducing the proliferation of satellite cells, yet at the opposite end of the spectrum, it contributes to muscle atrophy, inhibiting growth pathways^45^. This myokine can be released by muscle during the systemic stress response in ICU patients, where synergy with other inflammatory mediators upregulates muscle wasting^42,45^. Previous studies have shown a positive correlation of SMD in a population of critical patients and of IL-6 serum levels in a non-COVID ICU population^33^. Aschman et al.^46^, in a study evaluating skeletal muscle of COVID-19 patients at autopsy showed higher inflammation scores, characterizing mild-to-severe myositis. These authors also identified low or negative viral loads in most skeletal muscle analyzed; they suggested this was likely due to circulating virus rather than an infection of myocytes. Thus, myositis is probably associated with the systemic inflammation promoted by SARS-CoV-2^46^. This notion also corroborates with our hypotheses (summarized in Fig. 5) that the myosteatosis found in patients with COVID-19 may be due to the systemic acute inflammatory condition, and that skeletal muscle is another organ directly affected by the disease. Taken together, these data suggest myosteatosis might be a regular feature observed in patients with COVID-19, probably not only due to inflammation and insulin resistance caused by a pre-existing condition such as aging, diabetes and obesity (risk factors for severe COVID-19), but also additionally considering COVID-mediated inflammation (a common characteristic observed in this condition).

The increased neutrophil count reflects the intensity of systemic inflammation, whereas lymphopenia reflects lymphocyte sequestration at sites of inflammation and their apoptosis^47^. In SARS-CoV-2 infection, robust interferon suppression with lymphopenia is also found^48^. Therefore, NLR is a potential marker of the systemic inflammatory response, and has been used to predict the severity of COVID-19 and death^49–51^. In line with other reports, a high NLR was associated with poor outcomes in the present study. Patients had more clinical complications, such as acute distress syndrome, acute kidney injury, secondary infection, and shock. This marker also correlated significantly with death on univariate and multivariate analyses and prolonged LOS.

We devised a composite score based on SMD and NLR at diagnosis for use in clinical practice. The composite score predicted a poorer prognosis when both a low SMD and high NLR were associated. The Both group had significantly more complications (acute distress syndrome, acute kidney injury, secondary infection, shock, and ≥ 2 complications), as well as increased mechanical ventilation, LOS, and death. These results may reflect the relationship of disease severity and systemic inflammation in patients with COVID-19. These parameters can be readily assessed, and would help stratify patients at risk of poorer outcome at hospital admission. Furthermore, the composite score may represent a novel approach to guide studies in COVID-19 treatment, such as guiding the use of IL-6 receptor inhibitors in early stages of the disease in patients with both high NLR and low SMD.

The study has some limitations, including its retrospective design, lack of a control group (non-COVID-19 critically-ill patients), absence of an analysis of muscle mass changes over time, and the absence of serum samples for inflammatory marker measurements. It is also important to note that the length of the analysis, limited to 3 months of follow-up, may have been insufficient to capture long-term complications, as previously shown in studies of myosteatosis and muscle composition in non-COVID-19 critically ill patients, which demonstrated the importance of this biomarker in long-term survival, proving more marked than in acute outcomes^34^. Furthermore, no data was available in the present study on dietary intake, physical activity, socioeconomic status, or nutritional care support, factors which may well have affected SMI, SMD, and outcomes.

In the study cohort, 22.5% of patients died in the short-term. Consistent with previous studies, mortality rates in patients hospitalized due to COVID-19 in Brazil ranged from 21.7 to 47.3%^52,53^ versus mortality rates globally of 8 to 21% in patients hospitalized for SARS-CoV-2 pneumonia^54–57^. Therefore, the study results need to be further validated in other countries to increase generalizability.

## Conclusions

In the present study cohort, a low SMD (irrespective of being a pre-existing condition or a phenotype induced by COVID-19), high NLR, and a combination of these two pro-inflammatory factors, at diagnosis for SARS-Cov-2 infection predicted the severity of COVID-19. To our knowledge, this is the first study investigating the impact of both parameters on COVID-19 clinical outcomes. This new prognostic biomarker may help identify patients at risk for poorer outcomes at an early stage of COVID-19, and further studies are warranted to address its value as a predictive biomarker for COVID-19 therapeutic interventions.

## Data Availability

The original contributions presented in the study are included in the article/supplementary material, further inquiries can be directed to the corresponding author.
